# Firearm violence exposure and health in 2 national samples of Black and American Indian/Alaska Native adults

**DOI:** 10.1093/haschl/qxad036

**Published:** 2023-09-15

**Authors:** Daniel C Semenza, Nazsa Baker, Devon Ziminski

**Affiliations:** Department of Sociology, Anthropology, and Criminal Justice, Rutgers University, Camden, NJ 08102, United States; Department of Urban-Global Public Health, Rutgers University, Piscataway, NJ 08854, United States; New Jersey Gun Violence Research Center, Rutgers University, Piscataway, NJ 08854, United States; New Jersey Gun Violence Research Center, Rutgers University, Piscataway, NJ 08854, United States; New Jersey Gun Violence Research Center, Rutgers University, Piscataway, NJ 08854, United States; School of Social Work, Rutgers University, New Brunswick, NJ 08901, United States

**Keywords:** firearms, gun violence, health disparities, self-rated health, physical health, mental health

## Abstract

Exposure to firearm violence is widespread and disproportionately experienced by communities of color, with implications for broad health disparities. Survey data were collected from 2 nationally representative samples of Black (n = 3015) and American Indian/Alaska Native (AI/AN) (n = 527) adults in the United States in April and May 2023. The exposure measures were 4 types of firearm violence exposure. The outcome measures were self-rated health, number of poor physical health days, and number of poor mental health days. Regression results demonstrate that being threatened with a firearm and hearing about or witnessing a shooting were associated with poorer self-rated, mental, and physical health across both samples. Cumulative exposure to firearm violence was particularly associated with increasing harms to health for all outcomes. In general, individual and cumulative firearm violence exposures are linked to poorer health among Black and AI/AN adults in the United States. Significant enhancements and long-term investment are needed for firearm violence prevention to yield improvements to population health, particularly among communities burdened with high levels of exposure to firearm violence.

## Introduction

Firearm violence is a pressing public health problem in the United States. In 2021, nearly 48 000 people died as a result of a firearm injury.^1^ Firearm injuries are now the leading cause of death among children and adolescents ages 1 to 19 years old.^2,3^ Yet, exposure to firearm violence is not distributed equally throughout the US population. Young Black men are at the greatest risk of all racial groups for firearm homicide victimization and non-Hispanic American Indian and Alaskan Natives (AI/AN) also experience disproportionate rates of firearm homicide.^1^ Black Americans die at approximately 2.4 times the rate of their White counterparts from firearm violence, and in 2021, Black children were almost 13 times more likely to be killed in a firearm homicide than White children.^4^ The AI/AN firearm homicide rate is approximately 2.2 times higher than the rate for non-Hispanic White Americans.^4^ AI/AN women are at especially high risk for firearm homicide victimization linked to intimate partner violence compared with those in other racial groups. Yet, elevated firearm violence exposure in Black and AI/AN communities extends far beyond shootings where a person is killed. Nonfatal firearm injuries are estimated to outnumber firearm homicides in any given year by more than 2 to 1.^5^ These shootings disproportionately impact disadvantaged communities of color throughout the country.^6,7^

Exposure to general violence in one's community is associated with poorer mental and physical health.^8,9^ This association has implications for understanding how violence contributes to health disparities across racial and socioeconomic lines.^10,11^ Growing evidence suggests that specific exposure to firearm violence is particularly consequential for individual-level outcomes like anxiety and depression,^12^ suicidal ideation and psychotic experiences,^13^ and children's self-regulatory behavior and cognitive functioning.^14^ Communities with high rates of firearm violence similarly exhibit worse neighborhood-level outcomes, including poorer health behaviors and heightened rates of functional disability.^15,16^ Living in high-violence communities decreases the capacity to adequately exercise and sleep properly,^17,18^ while generating greater wear and tear on the body as a result of persistent stress exposure that exacerbates pre-existing health conditions and heightens risk for chronic ailments.^19,20^

There are numerous ways in which people are exposed to firearm violence that may be harmful to health. Direct victims of nonfatal shootings often suffer significant debilitation related to their injuries and must contend with chronic pain, functional limitations, and heightened risk for substance use.^21,22^ Those who have been injured or threatened with a firearm commonly experience symptoms of traumatic stress and feeling “shook.”^23^ Beyond personal experiences of threat and injury, those close to victims of firearm violence also experience substantial health harms. For instance, family members and friends of those who are shot and/or killed, especially youth, exhibit greater mental health needs after a shooting.^24,25^ Community outreach workers who regularly assist victims of firearm violence also experience elevated rates of mental distress and secondary trauma.^26^ Almost every American is now likely to know someone who has been a victim of firearm violence and this is particularly true among Black and AI/AN communities where rates of exposure are heightened.^27^

Finally, people may witness or hear about a shooting in their community but not personally know those involved. Exposure to community violence that entails living in violent residential environments and indirectly experiencing shootings is linked to a wide range of negative health outcomes.^8,9,28^ Taken together, people may experience direct exposure (being shot or threatened) and indirect exposure, which includes secondary (personally knowing a shooting victim) and community (witnessing/hearing about a shooting) exposures. These experiences are not necessarily mutually exclusive and, in fact, are likely to accumulate most among the very populations that experience the greatest levels of violence in their communities.

There remain significant gaps in understanding how firearm violence exposure is associated with health outcomes. First, research on firearm violence exposure and health often relies on relatively small, nonrepresentative samples and qualitative studies. Although this work is foundational, more research is needed that leverages nationally representative samples of those at greatest risk for firearm violence exposure. Second, there are no studies to our knowledge that examine how specific types of firearm violence exposure (eg, direct, secondary, community) are differentially associated with particular health outcomes. Finally, there remains a very limited understanding of how cumulative firearm violence exposure relates to health outcomes. Research suggests that people experience poly-victimization and exposure to different types of violence over time, which may be especially harmful to health.^29^ Yet, there has been little research to examine how cumulative exposure to different types of firearm violence corresponds to diverse health outcomes using nationally representative samples of those at elevated risk for exposure.

## Data and methods

### Data

We conducted a nationally representative survey of Black and AI/AN adults (18+ y) residing in the United States in April and May 2023. We focused specifically on these 2 populations given documented disproportionate exposure to firearm violence and poorer health outcomes compared with other racial groups.^6,30,31^ The surveys assessed a wide range of firearm-related behaviors, exposure to firearm violence, health outcomes, and demographic information. Surveys were disseminated by Ipsos Public Affairs, an international survey data-collection firm. The Black sample consisted of 3015 completed surveys while the AI/AN sample consisted of 527 completed surveys (completion rate: 59%). This study was approved by the International Review Board at Rutgers University.

Surveys were conducted using a sample from Ipsos’ KnowledgePanel, a probability-based web panel representative of the United States. Ipsos recruits panel members using address-based sampling to ensure full coverage of households in the United States. Based on the completion of an initial demographic survey to become a KnowledgePanel member, respondents received an email invitation to complete the present survey, which was administered in English. Email reminders were sent to nonresponders every 3 days after initial outreach. The median completion time of the survey was 27 minutes.

Post-stratification design weights were created for each sample of Black and AI/AN respondents to ensure national representativeness for the respective groups. The KnowledgePanel weighting methodology entails weighting the respondent pool to geodemographic benchmarks computed by combining the US Census Bureau's American Community Survey and the supplement of the Current Population Survey. A probability-proportional-to-size procedure was then used to select the study-specific samples and an iterative proportional fitting (raking) procedure was used to produce the final weights. For this study, KnowledgePanel design weights for qualified Black and AI/AN respondents were weighted to geodemographic distributions of gender, race, Census region, metropolitan status, education, and household income in each sample. See Appendix A for design weight benchmarks used in both samples. The resulting weights were trimmed and scaled to add up to the total number of qualified Black and AI/AN respondents.

### Measures

#### Outcomes

The self-rated health measure asked, “In general, how would you rate your health today?” Response categories included poor, fair, good, very good, and excellent. This global and validated measure predicts a wide range of health outcomes and mortality.^32^ The measure of physical health asked, “Thinking about your physical health, which includes physical illness and injury, for how many days during the past 30 days was your physical health not good?” The measure of mental health asked, “Thinking about your mental health, which includes stress, depression, and problems with emotions, how many days during the past 30 days was your mental health not good?” These 2 “health days” measures are regularly used in the Behavioral Risk Factor Surveillance System (BRFSS) and National Health and Nutrition Examination Survey (NHANES) run by the Centers for Disease Control and Prevention.^33^

#### Exposures

We measured lifetime firearm violence exposure types using 4 items, as follows:

1. “Threatened with a firearm” was measured with the question, “Have you ever been threatened with a firearm by another person?”
2. “Shot with a firearm” was measured using the question, “Have you ever been shot on purpose by another person with a firearm?”
3. We measured “family/friend shot” with the question, “Do you personally know someone, such as a friend or family member, who has been shot on purpose by another person with a firearm?”
4. Finally, we measured “witness/heard” about a shooting using the question, “Have you ever witnessed or heard about someone being shot intentionally by another person with a firearm in your neighborhood?”

Response categories for all 4 items were no/yes (reference = no). We measured “cumulative firearm violence exposure” by summing all 4 firearm violence exposure items to create a scale ranging from 0 through 4 (reference = 0). Given small cell sizes and wide confidence intervals in preliminary models due to low rates of respondents experiencing all 4 exposures (1.6% in the Black sample, 3.7% in the AI/AN sample), we collapsed the final category of the cumulative exposure index into “3 or more.”

#### Controls

All models controlled for a range of geodemographic variables in both samples, including sex (female, male), age (18–29, 30–44, 45–59, 60+ y), education (no high school, high school degree, some college, Bachelor’s or higher), household income (<$25 000, $25 000–74 999, $75 000–149 999, $150 000+), marital status (married, widowed, divorced, separated, never married), employment status (full time, part time, not working), health insurance (no, yes), metro area residence (no, yes), number of children living at home, and region of residence (Northeast, Midwest, South, West).

### Analytic strategy

We generated weighted descriptive statistics for all variables. We then conducted a series of regression models to examine the relationship between the 4 types of firearm violence exposure and 3 health outcomes controlling for all geodemographic measures. We conducted a second series of regression models to analyze the association between cumulative firearm violence exposure and each health outcome. We used ordinary least squares regression for the self-rated health models, given the outcome measure's normal distribution, and negative binomial regression for the physical and mental health days models, given the overdispersed count nature of the outcome variables. We used listwise deletion to account for a small number of missing cases in the Black (n = 126, 4%) and AI/AN (n = 17, 3%) samples.

## Results

Weighted descriptive statistics for all variables across both samples are shown in Table 1. Focusing on the main exposures, approximately 22% of Black respondents and 30% of AI/AN respondents reported having been threatened with a firearm. Approximately 3% of Black respondents and 6% of AI/AN respondents reported having been shot with a firearm. Beyond these direct exposures, approximately 41% of Black and 38% of AI/AN respondents reported knowing a family member or friend who had been shot. Approximately 38% of Black respondents and 28% of AI/AN respondents reported having witnessed or heard about a shooting in their neighborhood. The majority of respondents in both samples reported at least 1 type of firearm violence exposure (59% Black sample, 56% AI/AN sample). Cumulatively, approximately 12% of the Black sample and 13% of the AI/AN sample reported exposure to 3 or more types of firearm violence.

**Table 1.** Weighted descriptive statistics for Black (n = 3015) and AI/AN (n = 527) samples.

Measure	Black		AI/AN		Measure	Black		AI/AN	
	n	%	n	%		n	%	n	%
Self-rated health					Household income				
Poor	75	2	23	5	<$24 999	621	21	106	20
Fair	607	21	127	23	$25 000 to $74 999	1163	39	199	38
Good	1262	42	201	38	$75 000 to $149 999	831	28	156	30
Very good	789	27	135	26	$150 000+	399	13	66	12
Excellent	264	9	42	8	Marital status				
Firearm violence exposure types					Married	1079	36	256	49
Threatened w/firearm	649	22	159	30	Widowed	149	5	21	4
Shot w/firearm	80	3	33	6	Divorced	364	12	60	11
Family/friend shot	1237	41	201	38	Separated	66	2	12	2
Witnessed/heard about shooting	1138	38	147	28	Never married	1357	45	178	34
Cumulative firearm violence exposure					Employment status				
None	1230	41	231	44	Working full time	1596	53	240	46
One	789	27	135	26	Working part time	340	11	69	13
Two	604	20	90	17	Not working	1079	36	218	41
Three or more	347	12	68	13	Health insurance (y/n)	2697	90	448	85
Female	1646	55	280	53	Metro area residence	2756	91	396	75
Age (y)					Region of residence				
18–29	562	19	84	16	Northeast	513	17	44	8
30–44	972	32	192	36	Midwest	484	16	80	15
45–59	728	24	125	24	South	1700	57	192	36
60+	754	25	126	24	West	317	10	211	40
Education									
No HS	177	6	55	11		**Mean**	**SD**	**Mean**	**SD**
HS degree	1128	37	207	39	No. of days poor health in past month				
Some college	928	31	162	31	Physical	4.49	8.24	5.76	9.19
Bachelors or more	782	26	104	20	Mental	4.31	7.83	5.56	8.82
					No. of children living at home	0.64	1.07	0.67	1.20

Abbreviations: AI/AN, American Indian/Alaska Native; HS, high school.

Table 2 portrays the multivariate results for the association between each type of firearm violence exposure and the 3 health outcomes. In the Black sample, being threatened with a firearm (coefficient [coef.] = −0.200, *P* < .002) and witnessing/hearing about a shooting (coef. = −0.122, *P* < .026) were both significantly associated with poorer self-rated health. Similarly, being threatened with a firearm (coef. = −0.291, *P* < .05) and witnessing/hearing about a shooting (coef. = −0.295, *P* < .05) were each associated with poorer self-rated health in the AI/AN sample. With regard to physical health, witnessing/hearing about a shooting was associated with an increased rate of poor physical health days in the Black sample (incidence rate ratio [IRR] = 1.503, *P* < .001). On the other hand, being threatened with a firearm was associated with an increased rate of poor physical health days in the AI/AN sample (IRR = 2.710, *P* < .001). Finally, being threatened with a firearm (IRR = 1.338, *P* < .05) and witnessing/hearing about a shooting (IRR = 1.558, *P* < .001) were both associated with increased rates of poor mental health days in the Black sample. In the AI/AN sample, being shot was significantly associated with a higher rate of poor mental health days (IRR = 2.644, *P* < .05).

**Table 2.** Individual firearm violence exposures and self-rated health, physical health, and mental health.

	Self-rated health				Physical health days				Mental health days			
	Coef.	SE	*P*	CI	IRR	SE	*P*	CI	IRR	SE	*P*	CI
Black (n = 2889)												
Threatened	−0.200**	0.066	.002	−0.329, −0.071	1.149	0.152	.293	0.886, 1.490	1.338*	0.184	.034	1.022, 1.753
Shot	−0.084	0.195	.665	−0.466, 0.297	1.354	0.292	.159	0.888, 2.066	0.894	0.222	.652	0.550, 1.454
Family/friend shot	−0.062	0.056	.273	−0.172, 0.049	1.225	0.133	.061	0.990, 1.515	1.118	0.118	.288	0.910, 1.375
Witnessed/heard	−0.122*	0.055	.026	−0.230, −0.015	1.503***	0.171	.000	1.202, 1.878	1.558***	0.163	.000	1.268, 1.914
AI/AN (n = 510)												
Threatened	−0.291*	0.131	.027	−0.548, −0.033	2.710***	0.634	.000	1.711, 4.292	0.976	0.232	.918	0.611, 1.557
Shot	−0.281	0.235	.232	−0.742, 0.180	1.048	0.347	.888	0.547, 2.007	2.644*	1.060	.016	1.203, 5.810
Family/friend shot	−0.016	0.124	.895	−0.260, 0.227	1.472	0.325	.080	0.954, 2.271	1.014	0.250	.957	0.624, 1.647
Witnessed/heard	−0.295*	0.123	.017	−0.537, −0.052	1.337	0.247	.117	0.930, 1.922	1.315	0.282	.201	0.864, 2.003

Abbreviations: AI/AN, American Indian/Alaska Native; CI, confidence interval; Coef., coefficient; IRR, incidence rate ratio.

All models controlled for sex, age, education, household income, marital status, employment status, number of children living in the home, insurance status, metro area residence, and US region. ***P ≤ .001. **P ≤ .01. *P ≤ .05.

The results for the cumulative firearm violence exposure models are depicted in Table 3. For self-rated health, the magnitude of the negative association with firearm violence exposure grew as the number of exposure types increased in the Black sample (coefficients ranged from −0.152 for 1 type to −0.423 for ≥3 types). On the other hand, only cumulative exposure of 3 or more types was associated with poorer self-rated health in the AI/AN sample (coef. = −0.692, *P* < .001). For physical health, exposure to 2 (IRR = 1.551, *P* < .01) and 3 or more (IRR = 2.468, *P* < .001) types of firearm violence was associated with greater rates of poor physical health days among Black respondents. In the AI/AN sample, cumulative exposure was consistently associated with higher rates of physical health days at all levels of exposure (IRRs ranged from 2.802 for 1 type to 4.615 for ≥3 types). Finally, cumulative exposure was associated with higher rates of poor mental health days in the Black sample at all levels of exposure (IRRs ranged from 1.516 for 1 type to 2.408 for ≥3 types). Among AI/AN respondents, 3 or more exposure types wase associated with a higher rate of poor mental health days (IRR = 2.538, *P* < .001).

**Table 3.** Cumulative firearm violence exposure and self-rated health, physical health, and mental health.

	Self-rated health				Physical health days				Mental health days			
	Coef.	SE	*P*	CI	IRR	SE	*P*	CI	IRR	SE	*P*	CI
Black (Ref: none) (n = 2889)												
One	−0.152**	0.059	.010	−0.268, −0.036	1.273	0.159	.054	0.996, 1.627	1.516***	0.196	.001	1.177, 1.953
Two	−0.183**	0.067	.006	−0.314, −0.052	1.551**	0.218	.002	1.177, 2.044	1.604***	0.231	.001	1.209, 2.127
Three or more	−0.423***	0.083	.000	−0.593, −0.267	2.468***	0.417	.000	1.772, 3.437	2.408***	0.373	.000	1.777, 3.261
AI/AN (Ref: none) (n = 510)												
One	−0.159	0.133	.230	−0.420, 0.101	2.802***	0.674	.000	1.746, 4.496	1.671	0.448	.056	0.987, 2.830
Two	−0.262	0.152	.085	−0.561, 0.036	3.307***	0.848	.000	1.998, 5.474	0.645	0.179	.114	0.374, 1.112
Three or more	−0.692***	0.170	.000	−1.027, −0.357	4.615***	1.076	.000	2.919, 7.296	2.538***	0.713	.001	1.462, 4.407

Abbreviations: AI/AN, American Indian/Alaska Native; CI, confidence interval; Coef., coefficient; IRR, incidence rate ratio; Ref, reference.

All models control for sex, age, education, household income, marital status, employment status, number of children living in the home, insurance status, metro area residence, and US region.***P ≤ .001. **P ≤ .01. *P ≤ .05.

## Discussion

Structural racism and enduring systemic inequities have historically contributed to much greater violence exposure and poorer health among both Black and AI/AN populations in the United States. Black and AI/AN adults experience significantly higher rates of firearm violence exposure than their White counterparts.^4,6^ Each of these groups also have disproportionately higher rates of negative mental and physical health outcomes compared with other racial groups, especially White Americans.^30,31^ We argue that the enduring issue of firearm violence in America is also explicitly one of health equity. We set out to examine how individual and cumulative exposure to direct and indirect forms of firearm violence corresponds to self-rated, physical, and mental health outcomes using nationally representative samples of Black and AI/AN adults in the United States. Our results produced 3 key findings. First, more than half of all respondents in both samples reported at least 1 type of firearm violence exposure while a smaller, yet noteworthy, number reported 3 or more exposure types. Second, specific types of firearm violence exposure were associated with poorer health outcomes across samples. Being threatened with a firearm and witnessing/hearing about a shooting was most consistently associated with poorer health, while being shot and knowing a family/friend who had been shot were largely unrelated to the outcomes. Third, greater cumulative exposure to firearm violence was associated with poorer health across all outcomes in a largely linear fashion for both samples.

Our results signal important distinctions regarding type of firearm violence exposure and racial group in question when it comes to implications for health. In the Black sample, those directly threatened with a firearm were more likely to have poorer self-rated health while also experiencing higher rates of poor mental health days. In the AI/AN sample, being threatened with a firearm was associated with poorer self-rated health and a higher rate of poor physical health days. Being threatened with a firearm is a direct form of victimization that likely engenders significant fear and stress harmful to well-being.^19,20^ This may be especially salient for self-rated and physical health among AI/AN populations if firearms are brandished as threats against women in domestic violence–related altercations.^34^ The direct threat of violence may also inhibit one's ability to go outside in their neighborhood, engage in healthy behaviors (eg, obtain proper sleep, exercise regularly), and socialize regularly with others.^15–18^ Importantly, being shot was associated with poorer mental health in the AI/AN sample, suggesting that both forms of direct victimization have implications for certain types of healthin this group.

On the other hand, witnessing or hearing about a shooting in one's community was associated with poorer health across all outcomes in the Black sample, while only being linked to poorer self-rated health in the AI/AN sample. Since Black Americans are much more likely to live in dense urban settings where shootings regularly occur in local neighborhoods, these indirect experiences of firearm violence may be especially harmful to feelings of safety, security, and ultimately, well-being. Being aware of shootings near one's home may be a less common experience for AI/AN people, especially if they reside in relatively sparsely populated, rural areas where firearm violence occurs more often in the home than on the streets. In this way, geographical context may shape both how individuals are exposed to firearm violence and its implications for different health outcomes across racial groups.

It is notable that being shot and personally knowing someone who had been shot or killed were generally not associated with poorer health outcomes. This was somewhat surprising given prior evidence of the burdens for these types of exposures.^35,36^ The lack of significant findings for being shot may be attributed, in part, to a low base rate of those who report having experienced the specific exposure. Being shot or knowing someone who has been a victim of a shooting may also represent “acute” experiences of gun violence exposure, while the threat of violence or hearing about shootings in one's own neighborhood creates more looming, ongoing dangers that impart more significant damages to well-being for a longer period of time.^37^ The “lifetime” measurement of our exposure variables also means that the time between exposure and outcome could also be quite long, potentially reducing the influence of acute exposures, such as being shot or losing a loved one, on the health outcomes studied here.

Critically, we found that exposure to more types of firearm violence has significant implications for all health outcomes. This was particularly true in the Black sample, where we found evidence of a largely dose–response relationship between cumulative exposure and poorer health across dimensions. This linear association was also the case for poor physical health days among AI/AN respondents. However, cumulative exposure was only associated with self-rated health and poor mental health days in the AI/AN sample for 3 or more exposure types. This suggests a possible exposure threshold where 1 or 2 types do not influence certain aspects of health, but once significant exposure that combines both direct and indirect violence takes place, the association with poorer health becomes substantial. Over time, people living in communities with high rates of firearm violence are likely to be exposed to multiple instances and numerous types of firearm violence. Given that many Black and AI/AN communities throughout the country have endured high levels of firearm violence for decades, our results suggest that long-term, cumulative exposure may be especially damaging for collective health and well-being.

### Limitations and future research

There are certain limitations to this study. First, the cross-sectional nature of the data precludes causal claims regarding firearm violence exposure and health outcomes. The exposure measures are based on lifetime experiences with firearm violence while the outcome measures are based on current health or health in the past 30 days. Given the time anchors for these measures, we were unable to measure how recently the exposure to firearm violence occurred prior to completing the survey, and the strength of observed associations likely depends on the recency of exposure. We encourage researchers to use time-specific measures as well as a longitudinal design moving forward to properly assess the temporal association between firearm violence exposure and health.

Second, this study relied on self-report data, which are subject to response bias and recall error. The self-report nature of this study also captures a proximate range of individual forms of firearm violence exposure and does not account for exposure via traditional media consumption or social media use. Future work should consider linking personal exposures to firearm violence (either direct or indirect) to broader exposures via media sources. Third, despite efforts to control for pertinent demographic covariates, there are confounders that might influence the observed associations. For instance, neighborhood disadvantage, housing instability, and childhood adversities may be related to both the exposures and outcomes. Future studies should account for these potential confounders and others across the life course and at multiple levels of the social ecology.

Fourth, our study is limited to relatively broad health outcomes. Future research should explore associations of firearm violence exposure with a wider range health outcomes, including health behaviors (eg, sleep, exercise), functional disability and difficulties with daily activities, and specific psychological concerns (eg, suicidal ideation, posttraumatic stress disorder), where possible. Finally, our results can only be generalized to Black and AI/AN populations in the United States, both of which are disproportionately exposed to firearm violence. Future studies should assess the dynamics explored here among additional groups including Hispanic, White, and Asian populations to properly examine the extent of health disparities related to firearm violence exposure explored here.

### Policy implications

Our findings support greater efforts to directly address firearm violence exposure as a means of improving broader health outcomes, particularly among communities most affected by firearm violence.^6,7^ Conceptualizing firearm violence as a key driver of public health disparities supports continued efforts in violence intervention, prevention, and survivor services that focus on high-need populations. As such, we echo recent research that has called for the expansion of training for stakeholders in law enforcement, survivor advocacy, and health care systems to provide mental and physical health resources to specific populations in communities that experience high rates of firearm violence.^38^

Programs that support training for victim and survivor rights, foster collaborative efforts with local service provider networks, and offer financial support for violence intervention and prevention efforts are critical. One way to integrate these services is to centralize them through a local or state violence prevention office. For example, in 2022, the New Jersey Attorney General created a new division within the New Jersey Department of Law and Public Safety called the Division of Violence Intervention and Victim Assistance (VIVA) (https://www.njoag.gov/viva/). The VIVA office oversees victim/survivor services, violence intervention and prevention programming (eg, street outreach, hospital-based violence intervention programs), and staff health system and social service providers to directly reach community residents. Offices like VIVA bring together law enforcement, public health officials, community-based organizations, and other stakeholders to collaboratively reduce violence while providing resources to address health needs and mobilize financial support.

Offices of violence prevention can also support state and federal policies to increase health care access for those exposed to firearm violence. Recently, there have been efforts to use Medicaid funding to reimburse community violence programs.^39^ Dedicated offices for violence prevention programming alongside survivor services can foster a better-resourced infrastructure to address the complex mental and physical health needs of individuals exposed to firearm violence. Although these initiatives can expand the scope of the survivor populations served, it is essential that equitable access to initiative resources is prioritized, especially among communities with high levels of firearm violence exposure.

## Conclusion

This study highlights associations between firearm violence exposure and self-rated, physical, and mental health outcomes among 2 nationally representative samples of Black and AI/AN adults. Being threatened with a firearm and hearing about/witnessing a shooting appear to be particularly harmful to health, depending on the outcome and population in question. Cumulative firearm exposure was increasingly detrimental for all health outcomes in the study. The results cohere with a large body of research demonstrating that Black and AI/AN communities experience disproportionate rates of violence exposure and poor health outcomes. Exposure to firearm violence may have broad implications for public health that extend far beyond those that are directly victimized. As such, reducing firearm violence in America must be a top priority not only for its own moral sake but also as a means of improving well-being and health equity across racial groups throughout the country.

## Supplementary Material

qxad036_Supplementary_Data
