# Supplementary material for: Firearm violence exposure and health in 2 national samples of Black and American Indian/Alaska Native adults
Source: Health Aff Sch. 2023 Sep 15;1(3):qxad036. doi: 10.1093/haschl/qxad036 (PMC10986215; doi:10.1093/haschl/qxad036)
Supplement: qxad036_Supplementary_Data [file qxad036_Supplementary_Data.zip › GVEHealth_AppendixA.pdf]

Appendix A. 18+ African American Distribution Benchmarks for Design Weights

**Gender**	**Frequency**	**Percent**
**Male**	15975616	45.97
**Female**	18776282	54.03
**Age**	**Frequency**	**Percent**
**18-44**	18171478	52.29
**45-59**	8165840	23.5
**60+**	8414579	24.21
**Sex/Age**	**Frequency**	**Percent**
**18-44 Male**	8626014	24.82
**18-44 Female**	9545464	27.47
**45-59 Male**	3747000	10.78
**45-59 Female**	4418840	12.72
**60+ Male**	3602602	10.37
**60+ Female**	4811977	13.85
**Race**	**Frequency**	**Percent**
**African American only**	34358555	98.87
**Both**	393342.6	1.13
**Region**	**Frequency**	**Percent**
**Northeast**	5988446	17.23
**Midwest**	5617959	16.17
**South**	19544336	56.24
**West**	3601157	10.36
**MSA Status**	**Frequency**	**Percent**
**Non-Metro**	2901957	8.35
**Metro**	31849941	91.65
**Education**	**Frequency**	**Percent**
**LHS/HS**	15664113	45.07
**Some college**	10359643	29.81
**Bachelor or higher**	8728141	25.12
**Income**	**Frequency**	**Percent**
**Under $25,000**	6930017	19.94
**$25,000-$49,999**	7219110	20.77
**$50,000-$74,999**	6005875	17.28
**$75,000-$99,999**	4270889	12.29
**$100,000-$149,999**	5268908	15.16
**$150,000 and over**	5057099	14.55

18+ American Indian/Alaska Native Distribution Benchmarks for Design Weights

**Gender**	**Frequency**	**Percent**
**Male**	2362630	48.14
**Female**	2545445	51.86
**Age**	**Frequency**	**Percent**
**18-44**	2635470	53.7
**45-59**	1132415	23.07
**60+**	1140190	23.23
**Sex/Age**	**Frequency**	**Percent**
**18-44 Male**	1308107	26.65
**18-44 Female**	1327363	27.04
**45-59 Male**	571206	11.64
**45-59 Female**	561208.8	11.43
**60+ Male**	483316.7	9.85
**60+ Female**	656872.9	13.38
**Race**	**Frequency**	**Percent**
**Native American only**	4514732	91.99
**Both**	393342.6	8.01
**Region**	**Frequency**	**Percent**
**Northeast**	405395	8.26
**Midwest**	725628	14.78
**South**	1737154	35.39
**West**	2039898	41.56
**MSA Status**	**Frequency**	**Percent**
**Non-Metro**	1195168	24.35
**Metro**	3712906	75.65
**Education**	**Frequency**	**Percent**
**LHS/HS**	2510651	51.15
**Some college**	1462524	29.8
**Bachelor or higher**	934900	19.05
**Income**	**Frequency**	**Percent**
**Under $25,000**	957420.5	19.51
**$25,000-$49,999**	997862.8	20.33
**$50,000-$74,999**	813999.7	16.58
**$75,000-$99,999**	640131.8	13.04
**$100,000-$149,999**	777679.3	15.84
**$150,000 and over**	720980.2	14.69
